# Profitieren Schmerztherapiepatienten von ihren Mitpatienten? Eine retrospektive Beobachtungsstudie zum Einfluss des Stadiums der Schmerzbewältigung und der Therapieerfahrung der Mitpatienten auf den individuellen Therapieerfolg in einer stationären interdisziplinären multimodalen Schmerztherapie (IMST)

**DOI:** 10.1007/s00482-023-00774-x

**Published:** 2023-12-18

**Authors:** Nicole Lindenberg, Anika Bundscherer, Christoph Wiese, Christoph Lassen

**Affiliations:** 1 Zentrum für interdisziplinäre Schmerztherapie, Klinik für Anästhesiologie, Universitätsklinikum Regensburg, Franz-Josef-Strauß-Allee 11, 93053 Regensburg, Deutschland; 2 Klinik für Anästhesiologie und Intensivmedizin, Herzogin Elisabeth Hospital, Leipziger Straße 24, 38124 Braunschweig, Deutschland

**Keywords:** Chronischer Schmerz, Gruppenpsychotherapie, Patienteninteraktion, Behandlungserfolg, Transtheoretisches Modell, Chronic pain, Group psychotherapy, Patient interaction, Treatment outcome, Transtheoretical model

## Abstract

**Hintergrund:**

Interdisziplinäre multimodale Schmerztherapie (IMST) wird meist im Gruppensetting durchgeführt, um den Erfahrungsaustausch zwischen Patienten anzuregen und so die Veränderung schmerzbezogener Einstellungen und Verhaltensweisen zu erleichtern. Wie aus der Psychotherapieforschung bekannt ist, haben die Mitpatienten einer Therapiegruppe einen relevanten Einfluss auf den Therapieerfolg der einzelnen Patienten.

**Ziel der Arbeit:**

Wir untersuchten, inwieweit der Therapieerfolg in einer IMST-Gruppe von einzelnen Mitpatientencharakteristika wie dem mittleren Stadium kognitiv-verhaltensorientierter Schmerzbewältigung der Mitpatienten, der Differenz zum eigenen Schmerzbewältigungsstadium und dem Anteil an therapiewiederholenden Mitpatienten beeinflusst wird.

**Methodik:**

In einer retrospektiv geplanten Untersuchung der psychometrischen Testungen aller Patienten in einer stationären IMST zwischen Januar 2013 und Februar 2020 wurde mittels binärer logistischer Regressionsanalysen der Einfluss der Mitpatientencharakteristika auf klinisch relevante Veränderungen hinsichtlich verschiedener Parameter zur Ausprägung der chronischen Schmerzerkrankung analysiert.

**Ergebnisse:**

Untersucht wurden 540 Erstaufenthalte von 636 Behandlungsfällen. Pro Behandlungstag waren durchschnittlich 5 Mitpatienten, davon 15 % Therapiewiederholer, anwesend. Es zeigte sich, dass die Wahrscheinlichkeit, einen Erfolg in mindestens einem der untersuchten Parameter zu erreichen, zum einen vom Schmerzbewältigungsstadium der Mitpatienten (*p* < 0,001; OR = 2,885) und zum anderen vom Anteil an therapiewiederholenden Mitpatienten (*p* < 0,001; OR = 1,032) signifikant erhöht wird. Ein Einfluss auf den Therapieerfolg in einem spezifischen Parameter konnte nicht nachgewiesen werden.

**Fazit:**

Trotz methodischer Limitationen legen unsere Ergebnisse nahe, in Patientengruppen einer IMST therapieerfahrene Patienten und solche in einem fortgeschrittenen Schmerzbewältigungsstadium mit Neulingen und Patienten, die noch am Anfang der Bewältigung der Schmerzerkrankung stehen, zu kombinieren.

**Graphic abstract:**

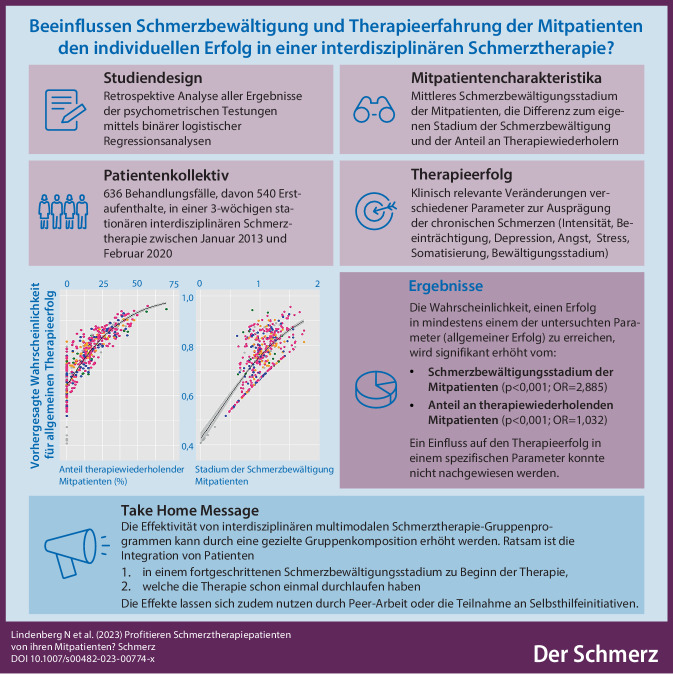

## Abstract (en)

**Background:**

Interdisciplinary multimodal pain therapy (IMPT) is mostly run in a group setting to encourage the exchange of experiences between patients and thus facilitate the change of pain-related attitudes and behavior. As is known from psychotherapy research, the fellow patients in a therapy group have a relevant influence on the success of the therapy for the individual patient.

**Objective:**

We examined the extent to which therapy success in an IMST group is influenced by individual co-patient characteristics, such as cognitive behavioral pain management, the difference to their own pain management and the proportion of co-patients who repeat therapy.

**Method:**

In a retrospectively planned investigation of the psychometric tests of all patients in an inpatient IMST between January 2013 and February 2020, the influence of fellow patient characteristics on clinically relevant changes with respect to various parameters of the severity of chronic pain disorders was analyzed using binary logistic regression analyses.

**Results:**

We examined 636 treatment cases of which 540 were first-time stays. On each day of treatment, 5 fellow patients were present, 15% of whom had repeated the therapy. We were able to show that the proportion of fellow patients who repeat the therapy (*p* < 0.001; odds ratio, OR = 1.032) and the cognitive behavioral pain management of the fellow patients (*p* < 0.001; OR = 2.885) significantly increase the probability of achieving success in at least one of the parameters examined. An influence of a specific parameter on the success of therapy could not be proven.

**Conclusion:**

Despite methodological limitations our results suggest that in patient groups of an IMST, patients with therapy experience and those with advanced cognitive behavioral methods for pain management should be combined with novices and patients who are still at the beginning of coping with the chronic pain disorder.

## Einleitung

Die einzelnen Behandlungselemente der interdisziplinären multimodalen Schmerztherapie (IMST) finden zumeist in Gruppen statt, um den Austausch zwischen Patienten anzuregen und die Veränderung schmerzbezogener Einstellungen und Verhaltensweisen zu erleichtern. Aus der Psychotherapieforschung ist bekannt, dass unter anderem die Gruppenzusammensetzung einen relevanten Faktor für den Therapieerfolg darstellt. Unklar ist noch, inwieweit die Mitpatienten, v. a. deren Krankheitsbewältigung und Therapieerfahrung, auch in einer multimodalen Schmerztherapie den individuellen Therapieerfolg mitbeeinflussen.

Für chronische Schmerzpatienten stellt die interdisziplinäre multimodale Schmerztherapie (IMST) eine der besten aktuell verfügbaren Behandlungsmöglichkeiten dar [49], auch wenn für eine größere wissenschaftliche Evidenz umfangreichere und strengere RCT gefordert werden [13]. Ihr Nutzen konnte speziell für Rückenschmerzen [28], Fibromyalgie [18], Kopf- [17] und Nackenschmerz [5] nachgewiesen werden. Charakteristisch für die IMST sind ein eng kooperierendes interdisziplinäres Behandlungsteam und das integrierte Zusammenwirken verschiedener somatischer, psychotherapeutischer und bewegungstherapeutischer Behandlungsansätze auf Basis eines biopsychosozialen Modells der Schmerzerkrankung [1]. Anforderungen an Indikation, Qualifikationen der Teammitglieder, Therapieinhalte und -methoden sowie Rahmenbedingungen für die Umsetzung sind für das stationäre und das tagesklinische Setting beschrieben [40].

Die IMST zielt vor allem auf Veränderungen der maladaptiven Schmerzverarbeitung und des Schmerzverhaltens [6] ab und ihr Erfolg wird stark von der Veränderungsbereitschaft der Patienten beeinflusst [59]. Für die Steigerung der Interventionseffektivität in der Therapie chronischer Schmerzen findet häufig das transtheoretische Modell nach Prochaska und DiClemente [43] Anwendung, welches Verhaltensänderung als einen Prozess von unterschiedlichen, aufeinander aufbauenden Stufen (Sorglosigkeit, Bewusstwerdung, Vorbereitung, Handlung, Aufrechterhaltung und andauernde Aufrechterhaltung) versteht und den Stufen entsprechende förderliche Strategien zur Verhaltensänderung zuordnet [51].

Die IMST wird meist in Gruppen durchgeführt [35], denn abgesehen von der Kosteneffektivität deuten Studien auf die Überlegenheit des Gruppensettings gegenüber der Einzelbehandlung hin, vor allem im Hinblick auf langfristige Veränderungen [15]. Die Durchführung der psychotherapeutischen Behandlungselemente in der Gruppe beeinflusst die kognitive Schmerzverarbeitung und das Coping langfristiger und nachhaltiger [22]. Es unterstützt die persönliche Entwicklung und kann ein positives Schmerzerleben und gesunde Verhaltensweisen fördern [50]. Die Deutsche Schmerzgesellschaft empfiehlt ebenfalls das Gruppensetting [40], um Erfahrungen auszutauschen und alternative Verhaltensweisen und Beziehungsmuster zu entwickeln [1]. Während einer Krankenhausbehandlung bietet der Austausch unter den Patienten sowohl gegenseitige Unterstützung als auch den Austausch von Erfahrungen [24] und „in gruppentheoretischer Perspektive lässt sich eine gesamte Psychotherapiestation als eine Großgruppe bzw. als ‚Gesellschaft im Kleinen‘ begreifen“ [9].

Die Wirksamkeit gruppenpsychotherapeutischer Elemente wird bestimmt von den Wechselwirkungen zwischen der formalen Veränderungstheorie, Gruppenleiter- und Patientencharakteristiken, strukturellen Faktoren (z. B. Häufigkeit, Frequenz, Gruppengröße, Setting und Bedarf an Ko-Therapeuten) und Kleingruppenprozessen [7]. Die Gruppenzusammensetzung hat dabei einen entscheidenden Einfluss auf die Entwicklung einer günstigen Gruppenatmosphäre [31]. Homogene Gruppen ermöglichen die Erfahrung der Universalität des Leidens [42], und da sich der Gruppenzusammenhalt schneller entwickelt [63], wird für zeitlich begrenzte Gruppentherapien, wie das bei der IMST der Fall ist, eher eine homogene Gruppenzusammensetzung empfohlen [58]. Andererseits erleichtert eine gewisse Heterogenität der Gruppenmitglieder „stellvertretendes Lernen, Feedback und neue Identifikationen“ [58]. Demnach sind eine Mischung aus erfahren Gruppenmitgliedern und Neulingen [25] und „eine gewisse Heterogenität in der Krankheitsschwere oder bei den Bewältigungsstufen“ [56] als eher nützlich anzusehen. Ähnlich zu der Annahme, dass Schüler in Klassen mit begabten Schülern bessere Lernerfolge erzielen [57], könnte man einen besseren therapeutischen Effekt in Gruppen mit erfolgversprechenderen Patienten erwarten.

Mit der vorliegenden Studie versuchten wir herauszufinden, ob der Therapieerfolg eines individuellen Patienten, der zum ersten Mal ein IMST-Programm absolviert, von dessen Mitpatienten beeinflusst wird hinsichtlich (1) des Stadiums kognitiv-verhaltensorientierter Schmerzbewältigung der anwesenden Mitpatienten, (2) der Differenz zwischen dem Stadium der Schmerzbewältigung bei Therapiebeginn des einzelnen Patienten und dem seiner Mitpatienten und (3) des Anteils an Therapiewiederholern unter den im Behandlungszeitraum anwesenden Mitpatienten.

## Methodik

### Studiendesign, Behandlungsmethode und Datenerhebung

Bei der vorliegenden Beobachtungsstudie handelt es sich um eine retrospektive Untersuchung routinemäßig erhobener Daten aller Patienten des stationären IMST-Programms im Zentrum für interdisziplinäre Schmerztherapie am Universitätsklinikum Regensburg zwischen Januar 2013 und Februar 2020. Dabei wurden sowohl Erstaufenthalte als auch Therapiewiederholungen untersucht, die Hypothesen wurden aber ausschließlich an den Erstaufenthalten getestet, weil davon auszugehen ist, dass sich der Einfluss von Therapiewiederholern auf Patienten, die selbst die Therapie zum wiederholten Mal durchführen, anders darstellt als bei Neulingen.

Das drei- bis vierwöchige Programm für bis zu 7 Patienten erfüllt alle Kriterien des Prozedurenschlüssels für die multimodale Schmerztherapie OPS 8‑918 [55]. Die Patienten werden je nach Belegung auf drei Doppel- und ein Einzelzimmer verteilt. Therapieelemente, die im Gruppensetting durchgeführt werden, sind: an Werktagen täglich angebotenes medizinisches Training und Übungen im Bewegungsbad oder Nordic Walking, eine jeweils einmal wöchentlich stattfindende ärztlich, psycho- und physiotherapeutisch geleitete Psychoedukation und mehrmals wöchentlich angebotene Anleitungen zur progressiven Muskelentspannung nach Jacobson und zur inneren Achtsamkeit. Im Einzelsetting finden ärztliche, psycho- und physiotherapeutische Interventionen und Biofeedback statt. Von 46 wöchentlich im Schnitt angebotenen Therapieeinheiten à 30 min finden 28 in der Gruppe statt, was einem Anteil von ca. 61 % entspricht. Die Patienten verbringen also pro Woche durchschnittlich 13 h und 15 min in gemeinsamen Therapien plus die Zeit, die die Patienten in ihrer therapiefreien Zeit je nach eigenem Bedarf miteinander verbringen. Die stationäre Aufnahme wird individuell je nach Behandlungsbedarf und -wunsch der Patienten terminiert und das Behandlungsteam legt wöchentlich den individuellen Behandlungsplan und die -dauer fest. Somit kommen die Patienten zu unterschiedlichen Zeitpunkten zur Gruppe und verlassen diese wieder, d. h., sie beginnen und beenden die Therapie unabhängig voneinander, entsprechend einem halboffenen Gruppensetting [30]. Wenn die Behandlung einmal absolviert wurde, besteht im weiteren Verlauf für alle Patienten die Möglichkeit, die Therapie zu wiederholen. Dies kann zum Wiederauffrischen oder Festigen der erlernten Schmerzbewältigungsstrategien, aber auch bei Veränderung oder Verschlechterung der Schmerzsymptomatik, z. B. aufgrund von veränderten somatischen Befunden, neuen psychosozialen Belastungen oder einem Rückfall in alte, dysfunktionale Schmerzbewältigungsmuster, indiziert sein. Patienten, deren Erstbehandlung zu keinerlei Erfolg geführt hat, werden in der Regel kein weiteres Mal aufgenommen. Das Wiederholen bzw. Wiederauffrischen der Therapieinhalte ist, meist in Form von sog. Booster-Wochen, in vielen schmerzmedizinischen Einrichtungen üblich [23, 41, 54]. Patienten, die die Therapie zum wiederholten Mal absolvieren, durchlaufen den gleichen Aufnahmeprozess und werden in einer Gruppe gemeinsam mit den Neulingen behandelt.

Für jeden Patientenaufenthalt wurden Informationen aus der stationären Behandlungsdokumentation entnommen, um demografische und behandlungsbezogene Daten zu erheben. Aus den Informationen zu Aufnahme- und Entlassdatum der einzelnen Behandlungsfälle wurde für jeden einzelnen Fall tagesgenau ermittelt, an welchem Behandlungstag sich welche Mitpatienten ebenfalls in der Therapie befanden und daraus für jede individuelle Behandlung die Charakteristika der im Behandlungszeitraum anwesenden Mitpatienten berechnet. Somit ist jeder Behandlungsfall sowohl Teil der Zielpopulation (im Fall einer Erstbehandlung) als auch Mitpatient (bei Erst- und wiederholten Behandlungen) und liefert Informationen sowohl zum individuellen Therapieerfolg bei Erstbehandlung als auch zu den Charakteristika der Mitpatienten.

Des Weiteren wurden die Ergebnisse der psychometrischen Testungen, die per Selbstbeurteilungsfragebogen jeweils zu Therapiebeginn und -ende durchgeführt werden, analysiert. Hier wurde hauptsächlich die Verlaufsversion des Deutschen Schmerzfragebogens (DSF; [33]) verwendet. Sie erhebt die Schmerzintensität und das Beeinträchtigungserleben und nutzt das Quality-of-Life Impairment by Pain Inventory (QLIP; [34]) und die Depressions-Angst-Stress-Skalen (DASS; [37]). Die Schwere somatoformer Symptome auf kognitiver und Verhaltensebene wurde mittels Quantifizierungsinventar für somatoforme Syndrome (QUISS; [61]) gemessen. Darüber hinaus wurde die motivationale Bereitschaft, kognitiv-verhaltensorientierte Schmerzbewältigungstechniken zu erlernen bzw. einzusetzen, mit dem Freiburger Fragebogen – Stadien der Bewältigung chronischer Schmerzen (FF-STABS; [32]) erhoben. Aufgrund von organisatorisch und lizenzrechtlich begründeten Veränderungen der verwendeten Messinstrumente und des Dokumentationsprozesses während des Beobachtungszeitraums konnten nicht alle Parameter für alle Patienten gleichermaßen erhoben werden. In Tab. 1 sind die angewandten Messinstrumente mit Erhebungszeiträumen, Anzahlen der Items, Subskalen, Beispielitems, Antwortmöglichkeiten, Berechnung des Gesamtscores und ggf. Cut-offs zusammengefasst.

**Tab. 1:** Überblick über die angewandten Messinstrumente mit Erhebungszeiträumen, Anzahlen der Items, Subskalen, Beispielitems, Antwortmöglichkeiten, Berechnung des Gesamtscores und ggf. Cut-offs

	Erhebungszeitraum	Items	Skalen	Beispielitem	Antwortmöglichkeiten	Gesamtscore und Cut-off
*Schmerzintensität *[31]	Oktober 2014 bis Februar 2020	3	– Momentane Schmerzintensität – Durchschnittliche Schmerzintensität – Maximale Schmerzintensität	Geben Sie jetzt bitte Ihre durchschnittliche Schmerzstärke während der letzten 4 Wochen an	NRS 0 bis 10	100-Punkte-numerische Rating-Skala (NRS) aus 10-fachem Mittelwert der drei Skalen
*Beeinträchtigungserleben *[31]	Oktober 2014 bis Februar 2020	3	– Beeinträchtigung im Alltag – Beeinträchtigung bei Freizeitaktivität – Beeinträchtigung der Arbeitsfähigkeit	In welchem Maße haben die Schmerzen in den letzten 3 Monaten Ihren Alltag (Ankleiden, Waschen, Essen, Einkaufen etc.) beeinträchtigt?	NRS 0 bis 10	100-Punkte-numerische Rating-Skala (NRS) aus 10-fachem Mittelwert der drei Skalen
*Quality-of-Life Impairment by Pain Inventory (QLIP; *[32, 54])	Oktober 2014 bis Juli 2018	7	Allgemeines Wohlbefinden	Wie war Ihr allgemeines Wohlbefinden?	VAS −100 bis +100 Punkte	– Summenscore zwischen 0 und 40 Punkten (40 = am wenigsten betroffen) – Schwerwiegende Beeinträchtigung bei ≤ 20 Punkten; schwerste Störung bei ≤12 Punkten
			Schlafdauer	War Ihre nächtliche Schlafdauer …?	Ausreichend/nicht ausreichend	
			Dauerschmerz	Hatten Sie Dauerschmerzen?	Nein/ja	
			Einschränkung	Wurden Sie durch Ihre Schmerzen in Ihren Tätigkeiten und Bedürfnissen eingeschränkt?	Nein/ein wenig/deutlich/stark/fast völlig	
			Beeinträchtigung der Stimmung	Haben die Schmerzen Ihre Stimmung beeinträchtigt?	Ein wenig/deutlich/stark/sehr stark	
			Linderungsmöglichkeit	Haben die Schmerzen Ihre Stimmung beeinträchtigt?	Ein wenig/deutlich/stark/sehr stark	
			Sonstige Beschwerden	Hatten Sie sonstige Beschwerden?	11 vordefinierte Symptome	
*Depressions-Angst-Stress-Skalen (DASS; *[35])	Oktober 2014 bis Februar 2020	21	– Depression (7 Items) – Angst (7 Items) – Stress (7 Items)	Ich konnte überhaupt keine positiven Gefühle mehr erleben. (Depression) Ich fühlte mich einer Panik nahe. (Angst) Ich fand alles anstrengend. (Stress)	0 („traf gar nicht auf mich zu“) bis 3 („traf sehr stark auf mich zu oder die meiste Zeit“)	Skalensummen zwischen 0 und 21 Punkten – Cut-off Depression = 10 – Cut-off Angst = 6 – Cut-off Stress = 10
*Quantifizierungsinventar für somatoforme Syndrome (QUISS; *[57])	Oktober 2014 bis Juli 2018	18	– 17 Items zu somatoformen Symptomen – 1 Item zu 41 somatoformen Symptomen, unterteilt in 6 Untergruppen	Haben Sie sich in der letzten Woche Sorgen gemacht, dass hinter Ihren Beschwerden eine ernsthafte Krankheit steckt?	0 („trifft überhaupt nicht zu“) bis 5 („trifft ausgesprochen zu“)	Summenscore zwischen 0 und 76 Punkten – 0–6: grenzwertig – 7–17: leicht – 18–30: mäßig – 31–44: deutlich – 45–58: schwer – > 58: sehr schwer
*Freiburger Itembogen – Stadien der Bewältigung chronischer Schmerzen (FF-STABS; *[30])	Januar 2013 und Februar 2020	17	– Sorglosigkeit (5 Items) – Vorbereitung (4 Items) – Handlung (4 Items) – Aufrechterhaltung (4 Items)	Ich habe die ernsthafte Absicht, in naher Zukunft mit meinen Schmerzen anders als bisher umzugehen	1 („trifft überhaupt nicht zu“) bis 5 („trifft genau zu“)	Skala mit dem höchsten Skalenmittelwert als wahrscheinlichstes aktuelles Bewältigungsstadium

Nach bestmöglicher Korrektur aller Diskrepanzen und Ausreißer wurden die Daten pseudonymisiert, in einer Tabellenkalkulationsdatei (Microsoft Excel Version 16.0) gespeichert und die Variablen mithilfe selbstprogrammierter Makros automatisiert berechnet.

### Variablen

Demografische und behandlungsbezogene Daten, die erhoben wurden, waren: *Geschlecht, Alter *zum Behandlungsbeginn und *Behandlungsdauer *in Tagen. Als Baseline-Variablen wurden bei Behandlungsbeginn gemessen: die charakteristische *Schmerzintensität* und die schmerzbezogenen *Einschränkungen*, jeweils auf einer 100-Punkte-numerischen Rating-Skala (NRS), ob wahrscheinlich eine ausgeprägte Belastung durch *Depression, Angst* oder *Stress* vorlag, die Stufe der schmerzbezogenen Lebensqualität (*LQ*), die Ausprägung einer *somatoformen Störung* und das Stadium der *Schmerzbewältigung*.

Aus den Informationen darüber, ob sich die in den individuellen Behandlungszeiträumen anwesenden Mitpatienten zum ersten oder zum wiederholten Mal in der Therapie befanden, und über deren Schmerzbewältigungsstadium zu Behandlungsbeginn wurden für jeden Behandlungsfall die folgenden unabhängigen Variablen berechnet:

- Das *mittlere* Stadium der *Schmerzbewältigung *bei Therapiebeginn aller im Behandlungszeitraum anwesenden *Mitpatienten*
- Die *mittlere Differenz* zwischen dem Stadium der *Schmerzbewältigung* bei Therapiebeginn des einzelnen Patienten und dem der im Behandlungszeitraum anwesenden Mitpatienten
- Der prozentuale *Anteil an Therapiewiederholern* unter den im Behandlungszeitraum anwesenden Mitpatienten *(Wiederholer)*

Die abhängigen Variablen sollten den Therapieerfolg eines einzelnen Patienten in der IMST abbilden. Wir bewerteten die IMST als erfolgreich, wenn die Testwerte des Patienten am Ende der Behandlung im Vergleich zum Behandlungsbeginn eines der folgenden Ergebnisse zeigten:

- Eine relevante *Reduktion der Schmerzintensität* (um mindestens 20 Punkte auf der 100-Punkte-NRS)
- Eine relevante *Reduktion der *schmerzbedingten *Einschränkung* (um mindestens 20 Punkte auf der 100-Punkte-NRS)
- Eine *Reduktion* der Testwerte von einer wahrscheinlich ausgeprägten Belastung durch *Depression, Angst* oder *Stress* auf einen regulären Wert
- Eine *Verbesserung* der schmerzbezogenen Lebensqualität (*LQ*) von der schwersten oder schweren Stufe zu einer weniger schweren Stufe
- Eine *reduzierte* Ausprägung der *somatoformen Störung *um mindestens eine Stufe
- Eine *Verbesserung *des *Schmerzbewältigung*sstadiums um mindestens ein Stadium

Darüber hinaus definierten wir den Erfolg in mindestens einem der sechs genannten Aspekte als *allgemeinen Erfolg*. Alle abhängigen Variablen haben nur zwei Ausprägungen (Erfolg oder kein Erfolg) und sind damit dichotom.

### Datenanalyse

Statistische Berechnungen wurden mit dem SPSS Statistik Paket Version 25.0.0.2 (IBM Corporation, Armonk, New York, Vereinigte Staaten) durchgeführt. Mittels binärer Regression wurde der Zusammenhang zwischen den Charakteristiken der Mitpatienten (Prädiktorvariablen) und dem Therapieerfolg des einzelnen Behandlungsfalls (Kriteriumsvariablen) untersucht. Die Regressionskoeffizienten wurden mittels Maximum-likelihood-Methode iterativ (bis der Parameter sich weniger als 0,001 ändert oder maximal 20 Iterationen erreicht) geschätzt. Die Verbesserung der Vorhersagekraft des Modells im Vergleich zur Basisverteilung der Parameter wurde getestet, indem die Prädiktorvariablen im ersten Block und ggf. vorhandene Confounder im zweiten Block (hierarchisches Modell, alle Variablen wurden in einem Schritt ins Modell eingeschlossen) hinzugefügt und χ^2^-Tests (Omnibustests) durchgeführt wurden. Die Anpassungsgüte des Modells wurde mittels Hosmer-Lemeshow-Test geprüft und der Pearson-Korrelationskoeffizient r wurde eingesetzt, um Multikolinearität auszuschließen. Als Maß der Varianzaufklärung nutzten wir Nagelkerkes R^2^ und konvertierten den Wert in die Effektgröße f^2^ nach Cohen [10]. Die Identifikation von Confoundern erfolgte mittels verschiedener statistischer Tests auf Unabhängigkeit demografischer, behandlungsbezogener und Baseline-Variablen von Prädiktor- und Kriteriumsvariablen. Dafür wurden in Abhängigkeit von den entsprechenden Messniveaus t‑ oder Mann-Whitney-U-Tests, einfaktorielle Varianzanalysen (ANOVA), Kruskal–Wallis-H-Tests, χ^2^- oder Cramers-V-Tests und Korrelationstests nach Pearson (r) oder Spearman (ρ) angewandt. Das globale Signifikanzniveau wurde auf 0,05 gesetzt und mittels Bonferroni-Holm-Prozedur lokal adjustiert [19].

## Ergebnisse

Es wurden insgesamt 636 Behandlungsfälle in die Untersuchung eingeschlossen, davon 540 Erstaufenthalte (Zielpopulation) und 96 Therapiewiederholungen. Die Erstpatienten lernten während ihrer Aufenthalte zwischen 0 und 5 wiederholende Patienten kennen (M = 1,40; SD = 1,167) und verbrachten zwischen 0 und 100 % ihrer Therapiezeit mit mindestens einem therapiewiederholenden Patienten (M = 54,11; SD = 40,38). Pro Behandlungstag hatten die Patienten in ihrer Erstbehandlung Kontakt zu 0 bis 3 wiederholenden Patienten (M = 0,79; SD = 0,718). Insgesamt trafen 73,7 % der Patienten während ihres Erstaufenthalts mindestens einen wiederholenden Patienten. Es zeigten sich schwache Unterschiede zwischen Baseline-Variablen bei Erstaufenthalten und Therapiewiederholungen hinsichtlich der mittleren Schmerzintensität (*p* < 0,001, r = 0,190) und der Wahrscheinlichkeit für eine hohe Belastung durch Stress (*p* = 0,036; V = 0,094), jeweils bei Behandlungsbeginn. Ein moderater Unterschied zeigte sich hinsichtlich des Schmerzbewältigungsstadiums bei Behandlungsbeginn: Die Patienten, welche die Therapie zum wiederholten Mal absolvierten, wiesen ein signifikant fortgeschritteneres Schmerzbewältigungsstadium (*p* < 0,001, V = 0,298) auf. Betrachtet man die Therapieerfolge der wiederholenden Patienten bei deren Erstaufenthalten, zeigt sich, dass die zukünftigen Therapiewiederholer signifikant häufiger einen Therapieerfolg hinsichtlich Stressbelastung (χ^2^ (1) = 4,105; *p* = 0,043) und Schmerzbewältigung (χ^2^ (1) = 5,435; *p* = 0,020) erzielten als Patienten, die die Therapie nur einmal absolvierten. In den anderen Bereichen erzielten beide Gruppen vergleichbare Erfolgsraten. In Tab. 2 sind die Merkmale der Gesamtstichprobe und der beiden Subgruppen Erstaufenthalte und Therapiewiederholungen mit Drop-out-Rate (z. B. durch unvollständig oder fehlerhaft ausgefüllte Fragebögen und Eingabefehler) und den Ergebnissen der Tests auf signifikante Gruppenunterschiede (*p*-Werte und ggf. Effektstärke) dargestellt, in Tab. 3 die Erfolgsraten derjenigen Erstaufenthalte, bei denen später keine Wiederholung der Therapie stattfand, und solcher mit Therapiewiederholung in der Zukunft und die Ergebnisse der Tests auf signifikante Unterschiede (*p*-Werte und Effektstärke).

**Tab. 2:** Merkmale der Gesamtstichprobe und der beiden Subgruppen Erstaufenthalte und Therapiewiederholungen mit Drop-out-Rate und den Ergebnissen der Tests auf signifikante Gruppenunterschiede (*p*-Werte und ggf. Effektstärke)

	Drop-outs (%)	Gesamtstichprobe	Erstaufenthalte	Therapiewiederholungen	*p*-Werte (Effekt)
		*n* = 636	*n* = 540	*n* = 96	
**Alter **(*n* = 636), *Jahre*	0	51,66 (13,09)^b^	51,42 (13,37)^b^	53,02 (11,35)^b^	0,270^e^
**Behandlungsdauer **(*n* = 636), *Tage*	0	21,16 (5,69)	21,24 (5,48)^b^	20,69 (6,80)^b^	0,450^e^
**Geschlecht **(*n* = 636)					
Männlich	0	270	228 (42,2)^c^	42	0,780^i^
Weiblich		366	312 (57,8)^c^	54	
**Schmerzintensität **(*n* = 523) *(NRS 0–100)*	28 (5,4)	73 (63–83)^a^	73 (60–80)^a^	80 (70–87)^a^	< 0,001^f^ (r = 0,190)
**Einschränkungen **(*n* = 523) *(NRS 0–100)*	32 (6,1)	70 (57–80)^a^	70 (53–80)^a^	70 (60–80)^a^	0,288^f^
**Depression **(*n* = 523) *Cut-off ≥* *10*	27 (5,2)	252 (50,8)^c,d^	207 (50,5)^c,d^	45 (52,3)^c,d^	0,757^g^
**Angst **(*n* = 523) *Cut-off ≥* *6*	27 (5,2)	259 (52,2)^c,d^	213 (52,0)^c,d^	46 (53,5)^c,d^	0,795^g^
**Stress **(*n* = 523) *Cut-off ≥* *10*	27 (5,2)	296 (63,1)^c,d^	236 (57,6)^c,d^	60 (69,8)^c,d^	0,036^g^ (V = 0,094)
**LQ **(*n* = 362)					
Normal	18 (5,0)	57 (16,1)^c^	48 (17,0)^c^	9 (14,5)^c^	0,683^h^
Schwer		121 (35,2)^c^	101 (35,8)^c^	20 (32,3)^c^	
Sehr schwer		166 (48,3)^c^	133 (47,2)^c^	33 (53,2)^c^	
**Somatoforme Störung **(*n* = 362)					
Grenzwertig	22 (6,1)	3 (0,9)^c^	2 (0,7)^c^	1 (1,6)^c^	0,660^h^
Leicht		4 (1,2)^c^	4 (1,4)^c^	0 (0)^c^	
Mäßig		35 (10,3)^c^	31 (11,1)^c^	4 (6,5)^c^	
Deutlich		132 (38,7)^c^	109 (39,1)^c^	23 (37,1)^c^	
Schwer		128 (37,5)^c^	103 (36,9)^c^	25 (40,3)^c^	
Sehr schwer		39 (11,4)^c^	30 (10,8)^c^	9 (14,5)^c^	
**Schmerzbewältigung **(*n* = 636)					
Sorglosigkeit	75 (11,8)	84 (13,2)^c^	73 (15,5)^c^	11 (11,5)^c^	< 0,001^h^ (V = 0,298)
Vorbereitung		333 (52,4)^c^	304 (64,4)^c^	39 (40,6)^c^	
Handlung		124 (19,5)^c^	79 (16,7)^c^	45 (46,9)^c^	
Aufrechterhaltung		20 (3,1)^c^	16 (3,4)^c^	4 (4,2)^c^	

^a^Median (IQR)

^b^Mittelwert (SD)

^c^Häufigkeit (%)

^d^Referenzwert = „ja“ bzw. „erhöht“

^e^T‑Test

^f^Mann-Whitney-U-Test

^g^χ^2^-Test

^h^Cramers V

**Tab. 3:** Erfolgsraten derjenigen Erstaufenthalte, bei denen später keine Wiederholung der Therapie stattfand und solcher mit Therapiewiederholung in der Zukunft und die Ergebnisse der Tests auf signifikante Unterschiede (p-Werte und Effektstärke)

	Gesamtstichprobe	Einmalige Behandlungen	Zukünftige Therapiewiederholer	*p*-Werte (Effekt)
	*n* = 540	*n* = 474	*n* = 66	
Reduktion Schmerzintensität	87 (379, 23)^a,b^	80 (347, 23)^a,b^	7 (32, 22)^a,b^	0,879^c^
Reduktion Einschränkungen	66 (373, 18)^a,b^	62 (342, 18)^a,b^	4 (31, 13)^a,b^	0,465^c^
Reduktion Depression	115 (379, 30)^a,b^	104 (348, 30)^a,b^	11 (31, 35)^a,b^	0,516^c^
Reduktion Angst	69 (380, 18)^a,b^	65 (349, 19)^a,b^	4 (31, 13)^a,b^	0,428^c^
Reduktion Stress	122 (380, 32)^a,b^	107 (349, 31)^a,b^	15 (31, 48)^a,b^	0,043^c^ V = 0,104^d^
Verbesserung LQ	151 (266, 57)^a,b^	130 (236, 55)^a,b^	21 (30, 70)^a,b^	0,120^c^
Reduktion somatoforme Störung	162 (258, 63)^a,b^	144 (228, 63)^a,b^	18 (30, 60)^a,b^	0,737^c^
Verbesserung Schmerzbewältigung	161 (433, 37)^a,b^	138 (390, 35)^a,b^	23 (43, 53)^a,b^	0,020^c^ V = 0,112^d^
Allgemeiner Erfolg	344 (451, 76)^a,b^	304 (405, 75)^a,b^	40 (46, 87)^a,b^	0,072^c^

^a^Häufigkeit (*n*, %)

^b^Referenzwert = „ja“

^c^χ^2^-Test

^d^Cramers V

Das mittlere Schmerzbewältigungsstadium der Mitpatienten war eins, was dem Stadium der Vorbereitung entspricht. Die mittlere Differenz zwischen dem Schmerzbewältigungsstadium des einzelnen Patienten und dem seiner Mitpatienten betrug im Mittel 0,5 Stadien. Die Patienten durchliefen die Therapie mit durchschnittlich 5 Mitpatienten pro Behandlungstag und davon 15 % mit Therapiewiederholern. Am Ende der Behandlung zeigten die Testergebnisse in 23 % der Fälle eine relevante Reduktion der Schmerzintensität und in 18 % der Fälle eine relevante Reduktion der schmerzbedingten Einschränkung. In 30 % der Fälle normalisierte sich die Wahrscheinlichkeit für eine ausgeprägte Belastung durch eine Depression. Das wurde auch in 18 % der Fälle für Angststörungen und in 32 % für Stress beobachtet. Eine Verbesserung der schmerzbezogenen Lebensqualität wurde in 57 % der Fälle erreicht und in 63 % der Fälle zeigte sich eine reduzierte Ausprägung der somatoformen Störung. In 38 % der Fälle beobachteten wir eine Verbesserung des Schmerzbewältigungsstadiums. Insgesamt beobachteten wir bei 76 % der Behandlungen einen allgemeinen Erfolg. Für die meisten demografischen, behandlungsbezogenen und Baseline-Variablen war es möglich, die Hypothese der statistischen Unabhängigkeit von sowohl Prädiktor- als auch Kriteriumsvariablen anzunehmen. Lediglich für die Variablen Behandlungsdauer, Schmerzintensität, schmerzbezogene Einschränkung und Schmerzmanagement, jeweils zu Behandlungsbeginn, fanden wir einen statistisch signifikanten Zusammenhang mit einzelnen Variablen des Therapieerfolgs und gleichzeitig zu einzelnen Mitpatientencharakteristiken. Diese Variablen wurden dementsprechend als Confounder betrachtet und in die binären Regressionsanalysen eingeschlossen, um deren zusätzlichen Einfluss auf den Therapieerfolg zu berücksichtigen. In Tab. 4 sind detaillierte Informationen zu unabhängigen (Prädiktor-) und abhängigen (Kriteriums‑)Variablen, deren Ausprägungen, zentralen Tendenzen bzw. Häufigkeiten und Ergebnisse der Signifikanztests auf Unabhängigkeit demografischer, behandlungsrelevanter und Baseline-Variablen von Prädiktor- und Kriteriumsvariablen (*p*-Werte) für die Gruppe der Erstaufenthalte (*n* = 540) dargestellt.

**Tab. 4:** Informationen zu unabhängigen (Prädiktor-) und abhängigen (Kriteriums-)Variablen, deren Ausprägungen, zentrale Tendenzen bzw. Häufigkeiten und Ergebnisse der Signifikanztests auf Unabhängigkeit demographischer, behandlungsrelevanter und Baseline-Variablen von Prädiktor- und Kriteriumsvariablen (p-Werte) für die Gruppe der Erstaufenthalte (*n* = 540)

	Unabhängige (Prädiktor‑)Variablen			Abhängige (Kriteriums‑)Variablen								
Variablen	Mittlere Schmerzbewältigung der Mitpatienten (*n* = 539)	Mittlere Differenz Schmerzbewältigung zu den Mitpatienten (*n* = 539)	Anteil Therapiewiederholer unter den Mitpatienten (*n* = 539)	Reduktion Schmerzintensität (*n* = 379)	Reduktion Einschränkungen (*n* = 373)	Reduktion Depression (*n* = 379)	Reduktion Angst (*n* = 380)	Reduktion Stress (*n* = 380)	Verbesserung LQ (*n* = 266)	Reduzierte somatoforme Störung (*n* = 258)	Verbesserung Schmerzbewältigung (*n* = 433)	Allgemeiner Erfolg (*n* = 451)
*Ausprägungen*	*0–3*	*0–3*	*0–100* *%*	*Ja/nein*	*Ja/nein*	*Ja/nein*	*Ja/nein*	*Ja/nein*	*Ja/nein*	*Ja/nein*	*Ja/nein*	*Ja/nein*
**Zentrale Tendenz/Häufigkeit**	1,00 (0,86–1,19)^a^	0,46 (0,22–0,84)^a^	15,05 (14,01)^b^	87 (23,0)^c,d^	66 (17,7)^c,d^	115 (30,3)^c,d^	69 (18,2)^c,d^	122 (32,1)^c,d^	151 (56,8)^c,d^	162 (62,8)^c,d^	161 (37,2)^c,d^	344 (76,3)^c d^
	**Ergebnisse der Signifikanztests auf Unabhängigkeit (*p*-Werte)**											
**Alter **(*n* = 540)	0,233^l^	0,613^l^	0,656^k^	0,169^e^	0,540^e^	0,565^e^	0,683^e^	0,805^**e**^	0,023^e^	0,020^**e**^	0,185^e^	0,197^e^
**Behandlungsdauer **(*n* = 540)	0,960^l^	0,214^l^	0,009^k^	0,125^e^	0,921^**e**^	0,292^e^	0,319^e^	0,004^e^	0,039^e^	0,106^e^	0,001^e^	0,480^e^
**Geschlecht **(*n* = 540)	0,940^f^	0,292^f^	0,079^e^	0,211^f^	0,102^f^	0,705^f^	0,894^f^	0,098^f^	0,591^f^	0,659^f^	0,911^f^	0,301^f^
**Schmerzintensität **(*n* = 410)	0,068^l^	0,421^l^	0,028^l^	< 0,001^i^	0,003^i^	0,027^i^	0,370^i^	0,820^i^	0,045^i^	0,059^i^	0,043^i^	0,060^i^
**Einschränkungen **(*n* = 405)	0,131^l^	0,010^l^	0,405^l^	0,005^f^	0,001^f^	0,016^f^	0,355^f^	0,164^f^	0,163^f^	0,144^f^	0,176^f^	0,192^f^
**Depression **(*n* = 410)	0,452^f^	0,367^f^	0,618^e^	0,008^i^	0,425^i^	< 0,001^i^	< 0,001^i^	< 0,001^i^	0,003^i^	0,989^i^	0,112^i^	< 0,001^i^
**Angst **(*n* = 410)	0,560^f^	0,921^f^	0,528^e^	0,116^i^	0,799^i^	< 0,001^i^	< 0,001^i^	< 0,001^i^	0,133^i^	0,935^i^	0,005^i^	0,014^i^
**Stress **(*n* = 410)	0,241^f^	0,172^f^	0,423^e^	0,035^i^	0,839^i^	< 0,001^i^	< 0,001^i^	< 0,001^i^	0,040^i^	0,746^i^	0,519^i^	0,002^i^
**LQ **(*n* = 282)	0,067^h^	0,072^h^	0,426^g^	0,002^j^	0,618^j^	< 0,001^j^	0,611^j^	0,057^j^	< 0,001^j^	0,350^j^	0,135^j^	0,010^j^
**Somatoforme Störung **(*n* = 279)	0,575^h^	0,547^h^	0,252^g^	0,434^j^	0,508^j^	0,093^j^	0,142^j^	0,128^j^	< 0,001^j^	< 0,001^j^	0,287^j^	0,001^j^
**Schmerzbewältigung **(*n* = 472)	0,031^h^	< 0,001^h^	0,168^g^	0,072^j^	0,921^j^	0,003^j^	0,528^j^	0,180^j^	0,534^j^	0,173^j^	< 0,001^j^	0,002^j^

^a^Median (IQR)

^b^Mittelwert (SD)

^c^Häufigkeit (%)

^d^Referenzwert = „ja“

^e^T‑Test

^f^Mann-Whitney-U-Test

^g^Einfaktorielle Varianzanalyse (ANOVA)

^h^Kruskal-Wallis-H-Test

^i^χ^2^-Test

^j^Cramers V

^k^Korrelation nach Pearson r

^l^Korrelation nach Spearman ρ

In den Regressionsmodellen zeigte sich, dass die Charakteristiken der Mitpatienten (Anteil an Therapiewiederholern, mittleres Schmerzbewältigungsstadium der Mitpatienten und Differenz zwischen der individuellen Schmerzbewältigung und der der Mitpatienten) lediglich im Hinblick auf die Wahrscheinlichkeit, einen allgemeinen Therapieerfolg zu erreichen, die Vorhersagekraft des Regressionsmodells signifikant verbesserten (χ^2^ (3) = 19,055; *p* < 0,001). Dieser Effekt blieb nach Einschluss des Schmerzbewältigungsstadiums zu Behandlungsbeginn als Confounder bestehen (χ^2^ (6) = 33,846; *p* < 0,001). Die Prädiktorvariablen zeigten hingegen keine Vorhersagekraft für den Therapieerfolg hinsichtlich der einzelnen Parameter Schmerzintensität (χ^2^ (3) = 6,584; *p* = 0,086), schmerzbedingte Einschränkung (χ^2^ (3) = 3,486; *p* = 0,323), Belastung durch Depression (χ^2^ (3) = 11,167; *p* = 0,011), Angst (χ^2^ (3) = 2,065; *p* = 0,559) oder Stress (χ^2^ (3) = 3,300; *p* = 0,348), schmerzbezogene Lebensqualität (χ^2^ (3) = 4,011; *p* = 0,260), Ausprägung einer somatoformen Störung (χ^2^ (3) = 2,889; *p* = 0,409) und Schmerzbewältigungsstadium (χ^2^ (3) = 1,099; *p* = 0,777). Tab. 5 zeigt die Maße der Modelgüte für jede Kriteriumsvariable: die Ergebnisse der Omnibustests (Modell‑χ^2^) und Nagelkerkes R^2^ nach Einschluss der Prädiktorvariablen (Block 1) und der Confounder (Block 2), wenn nötig.

**Tab. 5:** Maße der Modelgüte für jede Kriteriumsvariable: die Ergebnisse der Omnibus-Tests (Modell‑χ^2^) und Nagelkerke‘s R^2^ nach Einschluss der Prädiktorvariablen (Block 1) und der Confounder (Block 2)

	χ^2^	df	*p*	α_i_	R^2^
**Block 1** (Einschluss Prädiktorvariablen)					
*Reduktion Schmerzintensität *(*n* = 373)	6,584	3	0,086	0,0500	0,026
*Reduktion Einschränkungen *(*n* = 371)	3,486	3	0,323	0,0100	0,015
*Reduktion Depression *(*n* = 359)	11,167	3	0,011	0,0062	0,043
*Reduktion Angst *(*n* = 380)	2,065	3	0,559	0,0250	0,009
*Reduktion Stress *(*n* = 380)	3,300	3	0,348	0,0125	0,012
*Verbesserung LQ *(*n* = 264)	4,011	3	0,260	0,0083	0,020
*Reduktion somatoforme Störung *(*n* = 258)	2,889	3	0,409	0,0167	0,015
*Verbesserung Schmerzbewältigung *(*n* = 363)	1,099	3	0,777	0,0071	0,004
*Allgemeiner Erfolg *(*n* = 435)	19,055	3	< 0,001	0,0056	0,065
**Block 2** (Einschluss Schmerzbewältigung bei Behandlungsbeginn als Confounder)					
*Allgemeiner Erfolg *(*n* = 435)	33,846	6	< 0,001	0,0050	0,113

*χ*^*2*^ Chi-Quadrat, *df* Freiheitsgrade, *α*_*i*_ lokales Signifikanzniveau nach Bonferroni-Holm, *R*^*2*^ Nagelkerkes R^2^

**Abb. 1:**
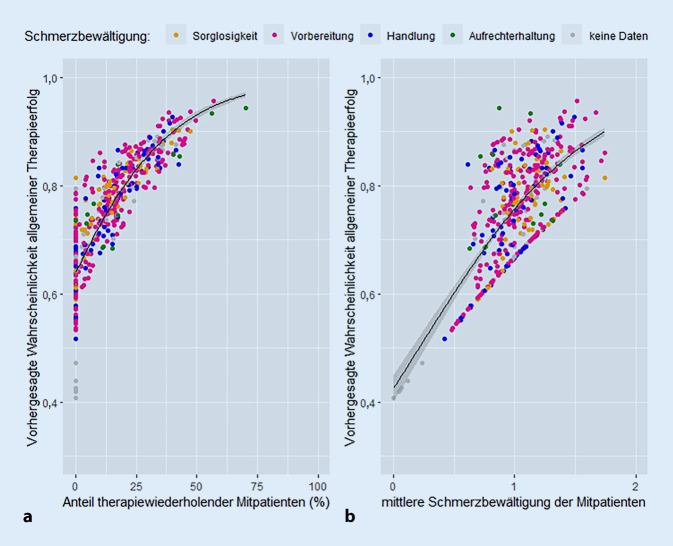
Vorhergesagte Wahrscheinlichkeit für einen allgemeinen Therapieerfolg in Abhängigkeit vom Anteil der therapiewiederholenden Mitpatienten (**a**) und dem mittleren Schmerzbewältigungsstadium der Mitpatienten (**b**), jeweils unter Berücksichtigung des Schmerzbewältigungsstadiums bei Behandlungsbeginn: Ergebnisse der binären logistischen Regressionsanalyse, dargestellt als Streudiagramm, einschließlich Regressionsgerade und Konfidenzintervall

Die Ergebnisse der binären logistischen Regressionsanalyse zeigten, dass die Wahrscheinlichkeit, einen allgemeinen Therapieerfolg zu erzielen, signifikant sowohl vom Anteil der Therapiewiederholer unter den Mitpatienten (OR = 1,032, 95 %-KI [1,012, 1,052]) als auch vom mittleren Schmerzbewältigungsstadium der Mitpatienten (OR = 2,885, 95 %-KI [1,042, 7,986]) beeinflusst wird. Die Schätzung der Regressionsparameter dieses Modells war stabil und Nagelkerkes R^2^ von 0,113 stellt ein akzeptables Maß an Varianzaufklärung [2] dar. f^2^ nach Cohen beträgt 0,127, was auf einen mittleren Effekt hindeutet [10]. Die Anpassungsgüte der Modelle war ebenfalls akzeptabel (Hosmer-Lemeshow-Test: *p* > 0,05) und aufgrund geringer Korrelationen zwischen den einzelnen Prädiktorvariablen (r < 0,85) kann Multikollinearität ausgeschlossen werden [52]. Detaillierte Ergebnisse der binären logistischen Regressionsanalysen zum Einfluss der Prädiktoren und Confounder auf die Chancen darauf, einen allgemeinen Erfolg zu erzielen, sind in Tab. 6 dargestellt. Abb. 1 zeigt den Anstieg der vorhergesagten Wahrscheinlichkeiten für das Erreichen eines allgemeinen Therapieerfolgs in Abhängigkeit vom Anteil an Therapiewiederholern unter den Mitpatienten (A) und dem mittleren Stadium der Schmerzbewältigung der Mitpatienten (B) unter Berücksichtigung des Stadiums der Schmerzbewältigung zu Beginn der Therapie.

**Tab. 6:** Ergebnisse der binären logistischen Regressionsanalysen zum Einfluss der Prädiktoren und Confounder auf die Chancen darauf, einen allgemeinen Erfolg zu erzielen

	Allgemeiner Erfolg (*n* = 435)				
	B	Wald	*p*	OR	95 %-KI
*Anteil therapiewiederholender Mitpatienten *(*n* = 539)	0,031	10,176	0,001	1,032	1,012–1,052
*Mittleres Schmerzbewältigungsstadium Mitpatienten *(*n* = 539)	1,059	4,160	0,041	2,885	1,042–7,986
*Mittlere Differenz individuelle und Mitpatienten-Schmerzbewältigung *(*n* = 539)	−0,501	0,800	0,371	0,606	0,202–1,815
*Schmerzbewältigung *(*n* = 472)	—	11,436	0,010	—	—
Schmerzbewältigung (1)	1,448	2,835	0,092	4,253	0,789–22,935
Schmerzbewältigung (2)	−0,104	0,011	0,918	0,901	0,123–6,592
Schmerzbewältigung (3)	−0,385	0,216	0,642	0,680	0,134–3,454
*Konstante*	0,27	0,001	0,982	0,973	—

*B* Regressionskoeffizient, *OR* Odds Ratio, *KI* Konfidenzintervall

## Schlussfolgerungen und Diskussion

Ziel dieser Untersuchung war es, zu zeigen, dass einzelne Mitpatientencharakteristiken in einer stationären IMST den individuellen Therapieerfolg beeinflussen. Konkret betrachteten wir den Einfluss der Anwesenheit von Patienten, welche die Therapie zum wiederholten Mal durchliefen, das Stadium der kognitiv-verhaltensorientierten Schmerzbewältigung der Mitpatienten und die Differenz zwischen der Schmerzbewältigung des einzelnen Patienten und der seiner Mitpatienten. Dafür analysierten wir retrospektiv routinemäßig erhobene Daten von Patienten in einem IMST-Programm, einschließlich Krankenhausdokumentation und Selbstbeurteilungsfragebögen zu Schmerzintensität, schmerzbedingten Beeinträchtigungen, Beeinträchtigungen durch eine Depression, Angst oder Stress, schmerzbezogener Lebensqualität, somatoformer Störung und Schmerzbewältigungsstadium. Es gelang uns nicht zu zeigen, dass die Gruppenzusammensetzung einen Einfluss auf einzelne Erfolgsparameter wie Schmerzreduktion, Reduktion schmerzbezogener Einschränkungen, Reduktion der Wahrscheinlichkeit für eine depressive, Angst- oder Stressstörung, Reduktion der Ausprägung einer somatoformen Störung, Verbesserung der Lebensqualität oder des Schmerzbewältigungsstadiums hat. Anders bei der Frage, ob Patienten einen Erfolg in mindestens einem der genannten Parameter, einen allgemeinen Erfolg, erreichten. Hier beobachteten wir die folgenden mittelgroßen Effekte: Steigt der Anteil der Therapiewiederholer unter den Mitpatienten oder das mittlere Schmerzbewältigungsstadium der Mitpatienten jeweils um eine Einheit an, erhöht sich die Wahrscheinlichkeit, einen Therapierfolg in mindestens einem Bereich zu erzielen, um den Faktor 1,032 bzw. 2,885, abhängig vom Stadium der Schmerzbewältigung zu Behandlungsbeginn. Das heißt, dass z. B. bei einem mittleren Schmerzbewältigungsstadium der Mitpatienten von 1 und einem Anteil an Therapiewiederholern von 15 % die Wahrscheinlichkeit auf einen Therapieerfolg bei ca. 82 % liegt, ohne Therapiewiederholer hingegen nur bei 74 %. Die Differenz zwischen der Schmerzbewältigung des einzelnen Patienten und der seiner Mitpatienten scheint hingegen keinen Einfluss auf den Therapieerfolg eines einzelnen Patienten zu haben.

Die Aussagekraft der vorliegenden Untersuchung wird durch verschiedene methodische Aspekte beeinträchtigt. Das retrospektive monozentrische Studiendesign ist einem prospektiven, multizentrischen, randomisierten, kontrollierten Experiment dahingehend selbstverständlich unterlegen. Zudem ist bekannt, dass in Beobachtungsstudien, bei denen Patienten eine individuelle Therapie erhalten, eine Vielzahl an unbekannten prognostischen Faktoren wirkt [53], wie z. B. die Unterbringung in Einzel- oder Mehrbettzimmern. Auch wenn die IMST insgesamt hinsichtlich ihrer Struktur im Rahmen des Prozedurenschlüssels der Deutschen Krankenhausgesellschaft standardisiert ist [55], führen unterschiedliche Therapeuten mit unterschiedlichen Eigenschaften die Therapie auf unterschiedliche Art und Weise durch. Diese sogenannten Therapeuteneffekte wirken besonders in Behandlungen mit größerem psychosozialem Schwerpunkt [26]. Außerdem birgt die Nutzung routinemäßig erhobener Daten ein hohes Risiko für Eingabefehler und die erwähnte Änderung der angewandten Messinstrumente beeinträchtigt die Datenqualität zusätzlich. Die verwendeten Selbstbeurteilungsbögen sind zwar alle validiert [32, 33, 61], aber die Durchführung der Messung war nicht standardisiert, was die Objektivität einschränkt [46]. Objektivere Messmethoden wie beispielsweise Verhaltensbeobachtungen oder die direkte Befragung der Patienten nach dem Einfluss ihrer Mitpatienten auf ihren eigenen Therapieerfolg könnten hier zu einem höheren Informationsgehalt der Untersuchung führen. An dieser Stelle kommt auch die Frage nach der bestmöglichen Objektivierung der therapeutischen Effekte einer IMST auf: Wären nicht Methoden der direkten Veränderungsmessung den indirekten Messungen mittels Vorher-nachher-Vergleich, so wie wir sie nutzten, vorzuziehen? Welche Outcome-Faktoren sind überhaupt relevant? Aktuell besteht ein Defizit bzgl. standardisierter Outcome-Parameter in IMST-Studien [12]. Bei der Frage, ab wann eine Schmerzreduktion als klinisch relevant zu betrachten ist, hielten wir uns an die wenigen verfügbaren Empfehlungen in der Literatur, wie z. B. in der Arbeit von Reinecke [44]. Schlussendlich entspricht unsere Stichprobe der üblichen Klientel in deutschen Schmerztherapiezentren [16], aber die große Zahl an Störvariablen beeinträchtigt die Interpretierbarkeit der Ergebnisse der binären logistischen Regression und die von uns gefundene Effektstärke ist auch eher klein.

Davon abgesehen entsprechen unsere Beobachtungen dem aktuellen Stand der Literatur, denn der Einfluss der Mitpatientencharakteristiken auf den individuellen Therapieerfolg konnte für verschiedene psychotherapeutische Gruppenprogramme schon gezeigt werden. Zum Beispiel fand die Arbeitsgruppe um Jill Paquin, welche die Veränderungen posttraumatischer Belastungssymptome in Traumatherapiegruppen analysierte, heraus, dass das Maß der Symptome aller Gruppenmitglieder vor der Behandlung einen positiven Einfluss auf die individuelle Veränderung der Traumasymptome der Patienten im Verlauf der Therapie hatte [38]. Auch Parker und Kollegen zeigten den Einfluss des Gruppenselbstbewusstseins auf das Outcome der einzelnen Patienten in einer kognitiv-verhaltenstherapeutischen Gruppentherapie [39]. Ansonsten wurde die Rolle der Mitpatientencharakteristika eher im Hinblick auf die Gruppenatmosphäre [11], den Gruppenzusammenhalt [8] oder die Beziehungserfahrungen zwischen den Patienten [48] untersucht. Die Frage nach dem Einfluss von Mitpatientencharakteristika auf den individuellen Therapieerfolg speziell in kognitiv-verhaltenstherapeutischen Gruppentherapien für chronischen Schmerz beantworten Diane Wilson und ihre Kollegen u. a. mit der Empfehlung, eine Mehrzahl an Patienten mit Entschädigungswunsch zu vermeiden [62], was unsere Ergebnisse unter der Annahme, dass Patienten mit Entschädigungswunsch eine geringere Veränderungsbereitschaft zeigen, ebenfalls unterstreicht.

Für die Praxis bedeuten unsere Ergebnisse, dass es in gruppenbasierten Schmerztherapieprogrammen empfehlenswerter erscheint, Patienten mit fortgeschrittener kognitiv-verhaltensorientierter Schmerzbewältigung in jede Gruppe einzuschließen (heterogene Zusammensetzung), anstatt Patienten in einem ähnlichen Schmerzbewältigungsstadium in einer Gruppe zusammenzubringen (homogene Zusammensetzung). Darüber hinaus scheint es ratsam, therapieerfahrene Patienten in die Therapiegruppe einzuschließen, wie das auch von Leszcz und Kobos empfohlen wird [25]. Das steht im Gegensatz zu den häufig angebotenen Booster-Wochen [47] zur Therapieauffrischung. Die Integration therapieerfahrener Patienten und solcher in einem fortgeschrittenen Schmerzbewältigungsstadium kann sowohl im offenen bzw. halboffenen (freiwerdende Plätze werden fortlaufend mit neuen Mitgliedern besetzt [30]) als auch im geschlossenen Setting (die Mitglieder beginnen und beenden die Behandlung, meist nach festgelegtem Zeitraum, auch gemeinsam [14]) erfolgen. Eine weitere Möglichkeit der praktischen Anwendung besteht darin, therapieerfahrene Patienten in Form von Peer-Arbeit in die Versorgung chronischer Schmerzpatienten mit einzubeziehen. Peer-Arbeit als therapeutisches Element wurde zuerst im Bereich der psychischen Gesundheitsfürsorge eingeführt, indem man Menschen, die selbst an einer psychischen Erkrankung litten, in die psychiatrische Behandlung oder Forschung mit einbezog [27]. Im Hinblick auf die Anwendung von Peer-Arbeit in der Schmerztherapie erscheinen die Forschungsergebnisse vielversprechend: Es konnten positive Effekte auf schmerzbezogene Selbstwirksamkeit, funktionelle Einschränkungen und Schmerzintensität [21], Opiatmissbrauchsrisiko [4] sowie kognitive Interferenz und Katastrophisieren [20] beobachtet werden. Der Gewinn besteht darin, zwischenmenschliche Verbindungen herzustellen, Zuspruch und Unterstützung auszutauschen und die Anwendung von Schmerzbewältigungsstrategien zu erleichtern [29]. Peer-Arbeit macht Hoffnung für die Zukunft und verbessert das eigene Stärkegefühl [45]. Sie ermutigt Patienten und informiert über Möglichkeiten für ein Leben nach der Therapie [60]. Auch die Teilnahme an Selbsthilfeinitiativen stellt eine Möglichkeit dar, Therapieerfahrungen der Patienten zur Unterstützung anderer Patienten zu nutzen. Diesbezüglich werden Gewinne für Selbstmanagementfähigkeiten, Krankheitsbewältigung und Krankheitswissen genauso wie die die Stärkung von Patienten in Arztgesprächen und die Reduktion der Krankheitsbelastung beschrieben [36]. Die Teilnahme an Selbsthilfeinitiativen kann eine Verbesserung der psychosozialen Befindlichkeit und der Hauptsymptome der jeweiligen Krankheit sowie die Verminderung von Bewegungseinschränkungen bewirken und dazu beitragen, Beziehungen positiver zu gestalten [3].

Weitere Untersuchungen mittels randomisierter, kontrollierter Experimente unter Verwendung objektiver Messinstrumente sind notwendig, um den Einfluss der Mitpatientencharakteristika auf den individuellen Therapieerfolg einzelner Patienten in der IMST zu untersuchen. Außerdem wäre die Rolle des subjektiv empfundenen Gruppenzusammenhalts und der Lernumgebung interessant, genauso wie ein direkter Vergleich der Erfolge in gemischten Therapiegruppen aus Neulingen und Therapiewiederholern mit der Anwendung von Booster-Sessions. Hier stellt sich auch die interessante Frage, ob sich für die Integration therapieerfahrener Patienten eine geschlossene oder eine offene Gruppenformation besser eignet.

## Fazit für die Praxis

Die Effektivität von interdisziplinären multimodalen Schmerztherapiegruppenprogrammen kann durch eine gezielte Gruppenkomposition erhöht werden. Ratsam sind:

- die Integration von Patienten in die Therapiegruppe, die die Therapie schon einmal durchlaufen haben,
- das Einschließen von Patienten mit einem fortgeschrittenen Schmerzbewältigungsstadium zu Beginn der Behandlung in jede Therapiegruppe und
- das Nutzen der Effekte durch die Implementierung von Peer-Arbeit sowie das Ermutigen der Patienten zur Teilnahme an Selbsthilfeinitiativen.

## References

[1] Arnold B, Brinkschmidt T, Casser H‑R et al (2014) Multimodale Schmerztherapie für die Behandlung chronischer Schmerzsyndrome. Schmerz 28(5):459–472 25216605 10.1007/s00482-014-1471-x

[2] Backhaus K, Erichson B, Plinke W et al (2018) Multivariate Analysemethoden. Springer, Berlin, Heidelberg

[3] Borgetto B (2002) Selbsthilfe im Gesundheitswesen. Bundesgesundheitsblatt Gesundheitsforschung Gesundheitsschutz 45(1):26–32 10.1007/s00103-001-0372-1 24676915

[4] Brooks JM, Umucu E, Storm M et al (2021) Preliminary outcomes of an older peer and clinician co-facilitated pain rehabilitation intervention among adults aged 50 years and older with comorbid chronic pain and mental health conditions. Psychiatr Q 92(2):561–571 32827098 10.1007/s11126-020-09831-5 PMC7897749

[5] Buchner M, Zahlten-Hinguranage A, Schiltenwolf M et al (2006) Therapy outcome after multidisciplinary treatment for chronic neck and chronic low back pain: a prospective clinical study in 365 patients. Scand J Rheumatol 35(5):363–367 17062436 10.1080/03009740600759795

[6] Bundesärztekammer (BÄK), Kassenärztliche Bundesvereinigung (KBV), Bundesärztekammer (BÄK), Kassenärztliche Bundesvereinigung (KBV), Arbeitsgemeinschaft der Wissenschaftlichen Medizinischen Fachgesellschaften (AWMF) (2017) Nationale VersorgungsLeitlinie Nicht-spezifischer Kreuzschmerz. www.kreuzschmerz.versorgungsleitlinien.de. Zugegriffen: 13. Mai 2022

[7] Burlingame GM, MacKenzie KR, Strauß B (2004) Small-Group-Treatment: Evidence for Effectiveness and Mechanisms of Chance. In: Lambert MJ (Hrsg) Bergin and Garfield’s handbook of psychotherapy and behavior change, 5. Aufl. Wiley, New York, S S. 647–S. 696

[8] Burlingame GM, McClendon DT, Alonso J (2011) Cohesion in group therapy. Psychother 48(1):34–42 10.1037/a0022063 21401272

[9] Caudill W (1958) The psychiatric hospital as a small society, 2. Aufl. Harvard University Press, Cambridge, Massachusetts

[10] Cohen J (1992) A power primer. Psychol Bull 112(1):155–159 19565683 10.1037//0033-2909.112.1.155

[11] Cropp C, Streeck-Fischer A, Jaeger U et al (2008) Der Zusammenhang zwischen Behandlungserleben und Behandlungserfolg bei der stationären Psychotherapie mit Kindern und Jugendlichen. Z Kinder Jugendpsychiatr Psychother 36(3):205–213 18622980 10.1024/1422-4917.36.3.205

[12] Deckert S, Kaiser U, Kopkow C et al (2016) A systematic review of the outcomes reported in multimodal pain therapy for chronic pain. Eur J Pain 20(1):51–63 26031689 10.1002/ejp.721

[13] Dragioti E, Evangelou E, Larsson B et al (2018) Effectiveness of multidisciplinary programmes for clinical pain conditions: An umbrella review. J Rehabil Med 50(9):779–791 30132012 10.2340/16501977-2377

[14] Eckert J (2007) Gruppenpsychotherapie. In: Reimer C (Hrsg) Psychotherapie. Springer, New York, S S. 651–S. 712

[15] Frettlöh J, Kröner-Herwig B (1999) Einzel- und Gruppentherapie in der Behandlung chronischer Schmerzen – Gibt es Effektivitätsunterschiede? Z Klin Psychol Psychother 28(4):256–266

[16] Frettlöh J, Maier C, Gockel H et al (2009) Patientenkollektiv deutscher schmerztherapeutischer Einrichtungen. Schmerz 23(6):576–591 19802633 10.1007/s00482-009-0836-z

[17] Gaul C, van Doorn C, Webering N et al (2011) Clinical outcome of a headache-specific multidisciplinary treatment program and adherence to treatment recommendations in a tertiary headache center: an observational study. J Headache Pain 12(4):475–483 21544647 10.1007/s10194-011-0348-y PMC3139052

[18] Häuser W, Bernardy K, Arnold B et al (2009) Efficacy of multicomponent treatment in fibromyalgia syndrome: a meta-analysis of randomized controlled clinical trials. Arthritis Rheum 61(2):216–224 19177530 10.1002/art.24276

[19] Holm S (1979) A simple sequentially rejective multiple test procedure. Scand J Stat 6(2):65–77

[20] Hruschak V, Rosen D, Tierney M et al (2021) Integrated psychosocial group treatment: a randomized pilot trial of a harm reduction and preventive approach for patients with chronic pain at risk of opioid misuse. Pain Med 22(9):2007–2018 33576415 10.1093/pm/pnaa461

[21] Khodneva Y, Richman J, Andreae S et al (2021) Peer support intervention improves pain-related outcomes among rural adults with diabetes and chronic pain at 12-month follow-up. J Rural Health 37(2):394–405 32124499 10.1111/jrh.12422 PMC9724177

[22] Kröner-Herwig B (2017) Schmerz als biopsychosoziales Phänomen – eine Einführung. In: Kröner-Herwig B, Frettlöh J, Klinger R et al (Hrsg) Schmerzpsychotherapie. Springer, Berlin, Heidelberg, S S. 3–S. 16

[23] Küchler A, Sabatowski R, Kaiser U (2012) Veränderungsmotivation bei Patienten mit chronischer Schmerzerkrankung nach einer multidisziplinären Behandlung. Die kurz- und langfristige Entwicklung. Schmerz 26(6):670–676 22972454 10.1007/s00482-012-1223-8

[24] Larsen LS, Larsen BH, Birkelund R (2014) A companionship between strangers—the hospital environment as a challenge in patient-patient interaction in oncology wards. J Adv Nurs 70(2):395–404 23829553 10.1111/jan.12204

[25] Leszcz M, Kobos JC (2018) Wie wissenschaftliche Evidenz praktisch genutzt werden kann: Gruppenpsychotherapie und die „Leitlinien für die klinische Praxis“ der American Group Psychotherapy Association (AGPA). In: Strauß B, Mattke D (Hrsg) Gruppenpsychotherapie. Springer, Berlin, Heidelberg, S S. 211–S. 224

[26] Lewis M, Morley S, van der Windt DAWM et al (2010) Measuring practitioner/therapist effects in randomised trials of low back pain and neck pain interventions in primary care settings. Eur J Pain 14(10):1033–1039 20444631 10.1016/j.ejpain.2010.04.002

[27] Mahlke C, Krämer U, Kilian R et al (2015) Bedeutung und Wirksamkeit von Peer-Arbeit in der psychiatrischen Versorgung. Nervenheilkunde 34(04):235–239

[28] Marin TJ, van Eerd D, Irvin E et al (2017) Multidisciplinary biopsychosocial rehabilitation for subacute low back pain. Cochrane Database Syst Rev 6(6):CD2193 28656659 10.1002/14651858.CD002193.pub2 PMC6481490

[29] Matthias MS, Kukla M, McGuire AB et al (2016) How do patients with chronic pain benefit from a peer-supported pain self-management intervention? A qualitative investigation. Pain Med 17(12):2247–2255 28025359 10.1093/pm/pnw138

[30] Mattke D, Schreiber-Willnow K (2002) Behandlung in geschlossenen versus halboffenen Gruppen in der stationären Psychotherapie. Gruppenpsychotherapie Gruppendynamik 38:153–172

[31] Mattke D, Strauß B (2018) Indikation, Prognose, Vorbereitung und Zusammensetzung von Therapiegruppen. In: Strauß B, Mattke D (Hrsg) Gruppenpsychotherapie. Springer, Berlin, Heidelberg, S S. 59–S. 67

[32] Maurischat C, Härter M, Auclair P et al (2002) Preliminary validation of a German version of pain stages of change questionnaire. Eur J Pain 6(1):43–48 11888227 10.1053/eujp.2001.0271

[33] Nagel B, Gerbershagen HU, Lindena G et al (2002) Entwicklung und empirische Überprüfung des Deutschen Schmerzfragebogens der DGSS. Schmerz 16(4):263–270 12192435 10.1007/s00482-002-0162-1

[34] Nagel B, Pfingsten M, Lindena G et al (2012) Deutscher Schmerz-Fragebogen – Handbuch. http://www.dgss.org/fileadmin/pdf/12_DSF_Manual_2012.2.pdf. Zugegriffen: 1. Juli 2013

[35] Nagel B, Pfingsten M, Brinkschmidt T et al (2012) Struktur- und Prozessqualität multimodaler Schmerztherapie. Schmerz 26(6):661–669 22956073 10.1007/s00482-012-1207-8

[36] Nickel S, Haack M, von dem Knesebeck O et al (2019) Teilnahme an Selbsthilfegruppen: Wirkungen auf Selbstmanagement und Wissenserwerb. Bundesgesundheitsblatt Gesundheitsforschung Gesundheitsschutz 62(1):10–16 30478487 10.1007/s00103-018-2850-8

[37] Nilges P, Essau C (2015) Die depressions-angst-stress-skalen. Schmerz 29(6):649–657 26205682 10.1007/s00482-015-0019-z

[38] Paquin JD, Kivlighan DM, Drogosz LM (2013) If you get better, will I? J Couns Psychol 60(2):171–179 23421778 10.1037/a0031904

[39] Parker TJ, Page AC, Hooke GR (2013) The influence of individual, group, and relative self-esteem on outcome for patients undergoing group cognitive-behavioural therapy treatment. Br J Clin Psychol 52(4):450–463 24117916 10.1111/bjc.12029

[40] Pfingsten M, Arnold B, Böger A et al (2019) Sektorenübergreifende interdisziplinäre multimodale Schmerztherapie. Schmerz 33(3):191–203 31073760 10.1007/s00482-019-0374-2

[41] Pöhlmann K, Tonhauser T, Joraschky P et al (2009) The Dachau multidisciplinary treatment program for chronic pain. Schmerz 23(1):40–46 18941803 10.1007/s00482-008-0727-8

[42] Pritz A (2001) Heterogene versus homogene Gruppenzusammensetzung. In: Tschuschke V, Agazarian YM (Hrsg) Praxis der Gruppenpsychotherapie. Thieme, Stuttgart, S S. 206–S. 208

[43] Prochaska JO, DiClemente CC (1984) Transtheoretical approach. Dow Jones-Irwin, New York

[44] Reinecke H (2010) Klinische Relevanz der therapeutischen Reduktion von chronischen nicht tumorbedingten Schmerzen. Logos-Verlag, Berlin

[45] Repper J, Carter T (2011) A review of the literature on peer support in mental health services. J Ment Health 20(4):392–411 21770786 10.3109/09638237.2011.583947

[46] Röhrig B, Du Prel J‑B, Blettner M (2009) Study design in medical research. Dtsch Ärztebl Int 106(11):184–189 19568374 10.3238/arztebl.2009.0184 PMC2695375

[47] Sabatowski R, Kaiser U (2012) Multimodal pain therapy. Schmerz 26(6):644–646 23183988 10.1007/s00482-012-1267-9

[48] Sammet I, Häfner S, Leibing E et al (2007) Perceived self-competence and relationship experiences in inpatient psychotherapy—a pilot study. Psychosoc Med 4:Doc04 19742295 PMC2736530

[49] Scascighini L, Toma V, Dober-Spielmann S et al (2008) Multidisciplinary treatment for chronic pain: a systematic review of interventions and outcomes. Rheumatol 47(5):670–678 10.1093/rheumatology/ken021 18375406

[50] Scherer EA, Scherer Z (2013) 1691—Group psychotherapy with people with chronic pain. Eur Psychiatry 28:121920709

[51] Schramm S (2006) Ein dynamischer Ansatz zur Steigerung der Veränderungsmotivation von chronischen Rückenschmerzpatienten. Eine prospektiv kontrollierte Interventionsstudie mit Messwiederholungsdesign. Dissertation, Universität, Fachbereich Psychologie, Hamburg

[52] Schroeder MA (1990) Diagnosing and dealing with multicollinearity. West J Nurs Res 12(2):175–184 2321373 10.1177/019394599001200204

[53] Schumacher M, Schulgen G (2002) Kontrollierte klinische Studien – eine Einführung. In: Schumacher M, Schulgen G (Hrsg) Methodik klinischer Studien. Springer, Berlin, Heidelberg, S S. 1–S. 20

[54] Schütze A, Kaiser U, Ettrich U et al (2009) Evaluation einer multimodalen Schmerztherapie am UniversitätsSchmerzCentrum Dresden. Schmerz 23(6):609–617 19756770 10.1007/s00482-009-0827-0

[55] Schwarze M, Hollo DF, Schiltenwolf M (2019) Assessment of the OPS code 8918 Multimodal pain therapy. Z Orthop Unfall 157(2):194–200 30290395 10.1055/a-0649-4971

[56] Schweiger U, Sipos V (2013) Gruppentherapie. Kohlhammer, Stuttgart

[57] Strauß B, Mattke D (2018) Gruppentherapieprozesse: Eine klinische Forschungsperspektive. In: Strauß B, Mattke D (Hrsg) Gruppenpsychotherapie. Springer, Berlin, Heidelberg, S S. 37–S. 57

[58] Tschuschke V (2003) Kurzgruppenpsychotherapie Theorie und Praxis. Springer, Wien

[59] Vlaeyen JWS, Morley S (2005) Cognitive-behavioral treatments for chronic pain: what works for whom? Clin J Pain 21(1):1–8 15599126 10.1097/00002508-200501000-00001

[60] von Wachter M, Hendrischke A (Hrsg) Psychoedukation bei chronischen Schmerzen. Springer, Berlin, Heidelberg

[61] Wedekind D, Bandelow B, Fentzahn E et al (2007) The quantification inventory for somatoform syndromes (QUISS): a novel instrument for the assessment of severity. Eur Arch Psychiatry Clin Neurosci 257(3):153–163 17203236 10.1007/s00406-006-0700-4

[62] Wilson D, Mackintosh S, Nicholas MK et al (2018) Are group size and composition associated with treatment outcomes in group cognitive behavioural therapy for chronic pain? Pain 159(4):783–792 29298215 10.1097/j.pain.0000000000001144

[63] Zielke M (1994) Zielsetzungen und Funktionen der Gruppentherapie in der stationären Behandlung. In: Zielke M (Hrsg) Handbuch Stationäre Verhaltenstherapie. Beltz, Weinheim, S S. 333–S. 343

